# Spatio-Trajectorial Optical Flow for Higher-Order Deformation Analysis in Solid Experimental Mechanics

**DOI:** 10.3390/s23094408

**Published:** 2023-04-30

**Authors:** Anna Bauer, Christoph Hartmann

**Affiliations:** Chair of Metal Forming and Casting, Technical University of Munich, Walther-Meissner-Strasse 4, 85748 Garching, Germany

**Keywords:** optical flow, variational methods, spatio-trajectorial, regularisation, strain rate, motion estimation

## Abstract

Material models are required to solve continuum mechanical problems. These models contain parameters that are usually determined by application-specific test setups. In general, the theoretically developed models and, thus, the parameters to be determined become increasingly complex, e.g., incorporating higher-order motion derivatives, such as the strain or strain rate. Therefore, the strain rate behaviour needs to be extracted from experimental data. Using image data, the most-common way in solid experimental mechanics to do so is digital image correlation. Alternatively, optical flow methods, which allow an adaption to the underlying motion estimation problem, can be applied. In order to robustly estimate the strain rate fields, an optical flow approach implementing higher-order spatial and trajectorial regularisation is proposed. Compared to using a purely spatial variational approach of higher order, the proposed approach is capable of calculating more accurate displacement and strain rate fields. The procedure is finally demonstrated on experimental data of a shear cutting experiment, which exhibited complex deformation patterns under difficult optical conditions.

## 1. Introduction

Over the years, simulations have become an essential part of many analyses and designs in science, as well as industrial application [1]. They allow verifying first drafts and investigating more advanced designs using experiments on real prototypes for validation. To perform meaningful numerical analysis and simulations, it is important to describe the mechanical properties of the materials that are used as accurately as possible. Next to the generally valid balance equations, these constitutive laws govern the continuum mechanics initial-boundary-value problem. Hence, the constitutive laws have to be determined with respect to the material and circumstances at hand. As they form the core to describe the system’s constitutive behaviour, material models in continuum mechanics become more complex, incorporating higher-order phenomena [2]. Hence, the extraction of higher-order motion information, i.e., the strain (In general, the strain represents a tensor-valued variable that describes the local deformation around a material point, ignoring rigid body motion.) and strain rate [2], or acceleration [3], from standard and complex motion patterns gains importance in the field of experimental mechanics. In particular, the robustness of evaluation methods to extract those fields from experimental data also under difficult conditions becomes important in this context [4,5,6]. These issues represent the main goal of the methods and investigations proposed in this paper.

## 2. State-of-the-Art

Currently, motion estimation and full-field deformation analysis in solid experimental mechanics almost entirely relies on digital image correlation (DIC) [7]. A group of methods based on the same basic assumptions as the DIC methods is the optical flow methods [8]. In contrast to DIC, these methods allow an adaption to the underlying problem by modifying the energy functional that serves as a basis for the calculation procedure [9]. While these methods have been applied to fluid mechanical problems [10,11], they are rarely adopted in structural and solid mechanics.

Hewer et al. [12], however, demonstrated a successful application of a higher-order variational approach on experimental data of a bi-axial tensile test able to estimate the displacements, as well as the strains. The choice of a higher-order regularisation term is particularly important not only to obtain more accurate displacement estimates, but in terms of the estimation of higher-order motion information, such as strain fields [13,14]. Since first-order regularised optical flow approaches are based on the assumption of vanishing displacement gradients, the corresponding strains will tend to zero instead of reflecting the underlying strains. This problem can be circumvented by choosing a spatial regulariser of at least second-order. Further, implementing the higher-order spatial regularisation in a purposeful manner as suggested by Hewer et al. [12], the displacement derivatives are directly reflected by the variational approach with no need for numerical derivation. Since this approach has been evaluated on a structural mechanics problem, it is used as a basis for the determination of the displacements and strains of a shear cutting experiment exhibiting complex deformation patterns.

In addition to the strains, also the strain rates occurring are to be determined. Hence, a regularisation in the temporal direction is to be integrated into the variational approach. Thus, additional information, which is available in the form of the further images of the video sequence, is incorporated in the variational approach, also allowing a more robust calculation of the strain and displacement fields.

The existing methods of temporal variation can generally be divided into two categories: temporal regularisation and trajectorial regularisation. While in the case of temporal regularisation, an assumption is made regarding the displacement gradient, in the case of trajectorial regularisation, it is the material point trajectory that is constrained [15,16,17].

Several approaches implementing some kind of temporal regularisation have been proposed [18,19,20,21,22]. However, the time derivatives of the smoothness term are not able to represent the actual trajectories for larger displacements, especially in the presence of motion boundaries [15]. In contrast to temporal regularisation, trajectorial regularisation is based on an assumption regarding the movement trajectory of the individual material point, similar to the Lagrangian description known from solid mechanics. A well-known approach of trajectorial regularisation is the procedure of Black and Anandan [23] registering the displacement field of the preceding time steps to the current time step in order to calculate smoothness in the trajectorial sense. Implementing not just a forward-pass of displacement information in time, like Black and Anandan [23], but an actual regularisation often results in complications regarding the optimisation process, since the displacement fields of different time steps refer to different coordinate systems. Hence, they have to be registered constantly anew to the coordinate system of the current time step [24]. Volz et al. [15] circumvented this downside by implementing a natural registration by a purposeful use of the parameterisation of the displacement fields.

The proposed spatio-trajectorial optical flow (STOF) model combines the spatial regularisation by Hewer et al. [12] and Volz et al. [15]. The following derivations and explanations were built on the results given in the respective references; see [12,15,25]. The results obtained using the proposed approach have already been published (see [2,26]), which supports its practical relevance. This paper contributes the mathematical derivation, technical background, and careful evaluation of the method.

## 3. Spatio-Trajectorial Optical Flow Model

This section discusses the modelling of the proposed optical flow energy functional, which is regularised both spatially and trajectorially. The presented approach combines the methods proposed by Hewer et al. [12] and Volz et al. [15].

In general, the energy functional:

(1) $$E=\int_{\Omega }E_{d}+E_{r}\;d\omega =\int_{\Omega }E_{d}+E_{s}+E_{t}\;d\omega ,$$

which must be minimised for the displacement calculation, consists of a data term $E_{d}$ and a regularisation term $E_{r}$, which commonly is of a spatial nature. The data term $E_{d}$ mostly implements the data-driven constraints of the brightness constancy assumption and gradient constancy assumption. $\Omega \subset R^{2}$ represents the region of interest in the image domain and dω the respective spatial increment.

**Figure 1:**
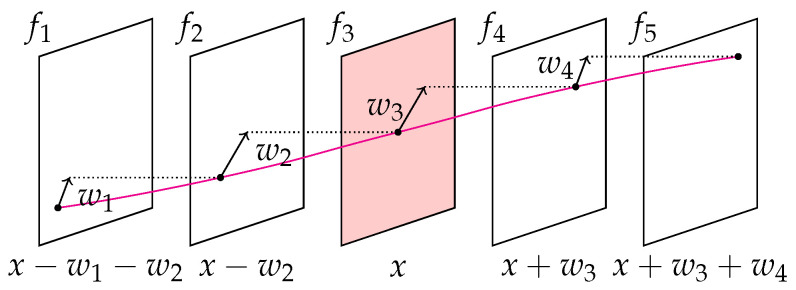
Trajectorial optical flow approach considering five successive images according to Volz et al. [15].

While the brightness constancy assumption $f_{1}\left(x,y\right)=f_{2}\left(x+u,y+v\right)$, often abbreviated as BCA, assumes that the individual material points (x,y) exhibit no change in their intensity value $f_{t}\left(x,y\right)$ with changing displacements w=(u,v), the gradient constancy assumption $\nabla f_{1}\left(x,y\right)=\nabla f_{2}\left(x+u,y+v\right)$, also referred to as GCA, copes with global illumination changes. Only implementing the BCA, no unique solution can be provided due to the so-called aperture problem. Hence, a regularisation term $E_{r}$ implementing certain assumptions, usually in the spatial domain, is added to the energy functional. Since both spatial and trajectorial smoothness shall be ensured, a trajectorial regularisation term $E_{t}$ is integrated into the energy functional in addition to the spatial regularisation term $E_{s}$. Therefore, more than two images need to be included in the variational approach. In order to implement a second-order trajectorial variation, at least four subsequent images need to be considered. Figure 1 visualises the principle of the trajectorial regularisation proposed by Volz et al. [15] considering five subsequent images.

The displacements $w_{i}=\left(u_{i},v_{i}\right),\;i\in \left\{1,2,3,4\right\}$ that occur between the five images are all given with respect to a common reference coordinate system, i.e., they are registered to the coordinate system of the reference frame $f_{3}$, in order to simplify the optimisation of the minimisation problem. Hence, the displacement $w_{1}$ can be considered as an increment added to $w_{2}$ in order to gain the trajectory from $f_{1}$ to $f_{3}$. The same accounts for $w_{4}$.

The modelling of the individual parts of the energy functional is discussed in more detail in the following subsections.

### 3.1. Data Term

As common with the optical flow approaches implementing a purely spatial variation, the data term of the STOF contains the BCA and GCA of the considered images. These assumptions can be formulated for two consecutive images $f_{i}$ and $f_{i+1}$. Therefore, considering five images $f_{i},i\in \left\{1,2,\ldots ,5\right\}$ simultaneously, as suggested by Volz et al. [15], four conditions $E_{d\;i,i+1}$ are needed to represent the relations between all neighbouring image pairs $f_{i}$ and $f_{i+1}$. Taken together, the four conditions yield the data term:

(2) $$E_{d}=E_{d12}+E_{d23}+E_{d34}+E_{d45}$$

of the energy functional that is to be minimised. Considering only the BCA (for reasons of better readability, but without loss of generality), the four conditions of the data term are calculated by

(3) $$\begin{array}{cc} E_{d12} & =\theta \cdot \Psi_{D}\left({|f_{2}\left(x-w_{2}\right)-f_{1}\left(x-w_{2}-w_{1}\right)|}^{2}\right)\\ E_{d23} & =\Psi_{D}\left({|f_{3}\left(x\right)-f_{2}\left(x-w_{2}\right)|}^{2}\right)\\ E_{d34} & =\Psi_{D}\left({|f_{4}\left(x+w_{3}\right)-f_{3}\left(x\right)|}^{2}\right)\\ E_{d45} & =\theta \cdot \Psi_{D}\left({|f_{5}\left(x+w_{3}+w_{4}\right)-f_{4}\left(x+w_{3}\right)|}^{2}\right)\;. \end{array}$$

The regularised $L_{1}$-norm $\Psi_{D}\left(s^{2}\right)=\sqrt{s^{2}+\varepsilon^{2}}$, ε>0, is used as a penalty function to make the variation procedure more robust. It is applied separately to the individual components $E_{d\;i,i+1}$ of the data term, since outliers can occur independently in the individual BCAs. Furthermore, a weighting factor θ=0.5 is introduced in order to weaken the influence of the two temporally more distant images. The GCAs are modelled accordingly. Combining both assumptions in the data components, for example given by

(4) $$\begin{array}{cc} E_{d23} & =\theta \cdot \left(\Psi_{D}\left({|f_{3}\left(x\right)-f_{2}\left(x-w_{2}\right)|}^{2}\right)\right)\\ \; & +\theta \cdot \left(\Psi_{D}\left({|\nabla_{2}f_{3}\left(x\right)-\nabla_{2}f_{2}\left(x-w_{2}\right)|}^{2}\right)\right), \end{array}$$

global illumination changes can be coped with. However, the data dependency can lead to a non-convex optimisation problem. Thus, the particularly desirable property of a unique minimum may no longer be given. Using the first-order Taylor approximation, it was assumed that the displacements to be calculated are small displacements, i.e., displacements smaller than 1 pixel [27]. For displacement calculations with larger displacements, so-called warping strategies are implemented [28,29].

### 3.2. Spatial Regularisation

Introducing trajectorial regularisation, four displacement fields $w_{1}$, $w_{2}$, $w_{3}$, and $w_{4}$ between the images $f_{j},\;j\in \left\{1,2,\ldots ,5\right\}$ were modelled by the data term $E_{d}$. In order to ensure the spatial smoothness of these displacement fields, the spatial regularisation emerges, similar to the data term, from the sum of the four individual components.

(5) $$E_{s}=\theta \cdot \left(E_{s1}+E_{s4}\right)+\left(1+\theta \right)\left(E_{s2}+E_{s3}\right)$$

Using the weighting factor θ, as already introduced in the data term, a weighting amplification was performed for the regularisation terms $E_{s2}$ and $E_{s3}$, while the influence of the terms $E_{s1}$ and $E_{s4}$ was attenuated. The subscript i∈{1,2,3,4} of the individual terms $E_{si}$ of the spatial regularisation term refers to the corresponding displacement field $w_{i}$, which is assumed to exhibit some kind of spatial smoothness. With these terms, a priori information about the spatial behaviour of the displacement fields $w_{i}$ is to be integrated into the variational approach. In this case, a third-order regularisation term as proposed by Hewer et al. [12] was used. Thus, the components of the regularisation term are defined by the generic rule:

(6) $$E_{si}=\alpha (||A_{i}-\nabla w_{i}{\left|\right|}_{2}^{2}+\alpha (||B_{i}-\nabla A_{i}{\left|\right|}_{2}^{2}+\alpha \left(S_{\Psi }\left(B_{i}\right)\right)))$$

for the individual displacement fields $w_{i},\;i\in \left\{1,2,3,4\right\}$. Therefore, the auxiliary variables $A=A_{i|kl},\;k,l\in \left\{1,2\right\}$ and $B=B_{i|klm},\;k,l,m\in \left\{1,2\right\}$ were introduced, which were set in relation to the displacements $w_{i}=\left(u_{i},v_{i}\right)$ in the so-called agreement terms $\left|\right|A_{i}-\nabla w_{i}{\left|\right|}_{2}^{2}$ and $\left|\right|B_{i}-\nabla A_{i}{\left|\right|}_{2}^{2}$. In the first agreement term $\left|\right|A_{i}-\nabla w_{i}{\left|\right|}_{2}^{2}$, the difference between the displacement gradient $\nabla w_{i}$ and the auxiliary variable $A_{i}$ is minimised in the sense of the quadratic Frobenius norm. Hence, the variable $A_{i}$ acts as an estimate of the displacement gradient $\nabla w_{i}$. The same can be observed for the second agreement term $\left|\right|B_{i}-\nabla A_{i}{\left|\right|}_{2}^{2}$, in which the difference between the gradient of $A_{i}$ and $B_{i}$ is minimised. Thus, by introducing the agreement terms with the auxiliary variables $A_{i}$ and $B_{i}$, a simultaneous determination of the displacement gradient $\nabla w_{i}$ is performed by the variable $A_{i}$, as well as the gradient of the displacement gradient $\nabla^{2}w_{i}$ by the variable $B_{i}$, besides the calculation of the displacements $w_{i}$. In the remaining term of the spatial regularisation:

(7) $$S_{\Psi }\left(B_{i}\right)=tr\;\left(\Psi \left(\sum_{k,l,m=1}^{2}\nabla B_{i|klm}\nabla B_{i|klm}^{T}\right)\right)\;,$$

the third-order smoothness assumption is formulated. This first-order smoothness assumption with respect to *B* corresponds to a second-order smoothness assumption with respect to *A* and, thus, to a third-order smoothness assumption with respect to the displacements *w*.

### 3.3. Trajectorial Regularisation

In addition to the spatial regularisation term, a trajectorial regularisation term as suggested by Volz et al. [15] is integrated into the energy functional, which makes an assumption regarding the displacement gradient in the temporal direction, i.e., the displacement rate. While the spatial regularisation was applied individually to the displacement fields $w_{1}$, $w_{2}$, $w_{3}$, and $w_{4}$, the trajectorial regularisation:

(8) $$\begin{array}{cc} \; & E_{t}=E_{t1}+E_{t2}\;\;\;\mathrm{with}\\ \; & E_{t1}=\Psi_{C}\left({\left(u_{3}-2u_{2}+u_{1}\right)}^{2}+{\left(v_{3}-2v_{2}+v_{1}\right)}^{2}\right)\;,\\ \; & E_{t2}=\Psi_{C}\left({\left(u_{4}-2u_{3}+u_{2}\right)}^{2}+{\left(v_{4}-2v_{3}+v_{2}\right)}^{2}\right)\;, \end{array}$$

binds a coupling of these four fields via second-order finite differences into the variational approach. Instead of the robust penaliser $\Psi_{D}$, as it was used in the data term, the Charbonnier penaliser $\Psi_{C}\left(s^{2}\right)=2\lambda^{2}\sqrt{1+\frac{s^{2}}{\lambda^{2}}}-2\lambda$ was used for the temporal regularisation. With the setting of the contrast parameter λ, a specific adjustment of the weighting of the residuals to the underlying image dataset is possible.

### 3.4. Optimisation

After having described the individual components of the proposed STOF energy functional in detail, the focus is now on its optimisation. The optimisation was realised by calculating the zeros of the respective Euler–Lagrange equations:

(9) $$\frac{d}{dt}\frac{\partial E}{\partial {\dot{q}}_{i}}-\frac{\partial E}{\partial q_{i}}=0.\;i=\left\{1,\ldots ,l\right\}\;,$$

of the energy functional, which is dependent on the variables $q_{i},\;i\in \left\{1,\ldots ,l\right\}$. By applying the calculation rule of the Euler–Lagrange Equation (9), a system of *l* differential equations results, which needs to be solved in order to determine the minimum of the energy functional [30]. For a third-order spatial variational approach and under the consideration of five temporally successive images, for the realisation of a second-order trajectory variation, 56 Euler–Lagrange equations contribute to the solution of the energy functional. There are 8 Euler–Lagrange equations that result from the displacement fields $w_{i}=\left(u_{i},v_{i}\right)$, 16 Euler–Lagrange equations from the corresponding displacement gradients $A_{i|kl},\;k,l\in \left\{1,2\right\}$, and 32 Euler–Lagrange equations from the gradients of the displacement gradients $B_{i|klm},\;k,l,m\in \left\{1,2\right\}$, which act as auxiliary variables in the spatial regularisation term. The index i∈{1,2,3,4} in $A_{i|kl}$ and $B_{i|klm}$ refers to the displacement field $w_{i}$, while the indices k,l,m∈{1,2} address the components of the auxiliary variables.

To apply the optimisation problem to image data, the Euler–Lagrange equations are discretised via finite differences. The discretised equations are to be solved in a system of equations analogous to Hewer [25].

## 4. Validation

**Figure 2:**
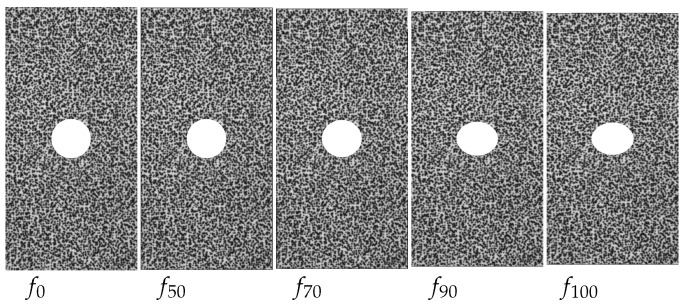
Sample images of the artificially generated validation data of a pressure test of a perforated tensile sample.

For the evaluation and performance classification of optical flow processes, benchmarks are used as the standard. Those provide different image sequences, which contain the currently prevailing difficulties of optical flow calculations. Among the best-known benchmarks in optical flow analysis are the Middleburry Benchmark [31] and the KITTI Benchmark [32], which was especially developed for autonomous driving. However, since the goal is not a universally applicable optical flow method, but rather, specifically aiming at the calculation of the displacements, strains, and strain rates occurring in a sheet metal sample, synthetically generated image sequences of a compression test of a perforated sheet metal sample were used instead of the above-mentioned benchmarks. This test has already been successfully used for validation purposes, and for a detailed description, refer to [33]. The introduction of a time scale was omitted for the artificial data, so that they remain without units. An excerpt of the synthetic image data that were used is depicted in Figure 2.

**Figure 3:**
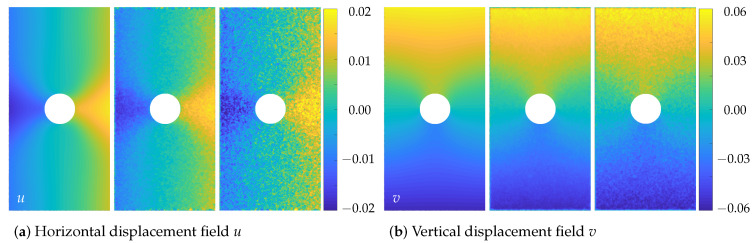
Horizontal displacement-field *u* (**a**) and vertical displacement field *v* (**b**) between the images $f_{20}$ and $f_{30}$. **Left**: ground truth, **Middle**: STOF approach, **Right**: spatial OF approach.

**Figure 4:**
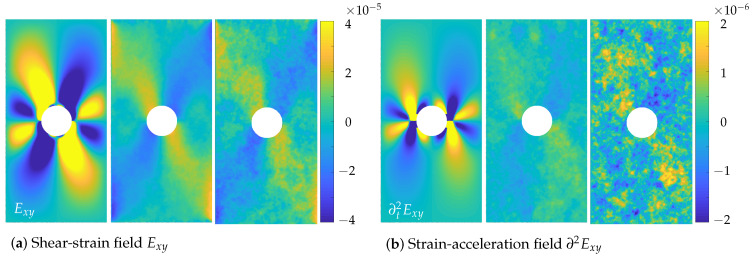
Shear-strain-field $E_{xy}$ (**a**) between the images $f_{20}$ and $f_{30}$ as well as strain acceleration field $\partial^{2}E_{xy}$ (**b**) between the images $f_{20}$, $f_{30}$ and $f_{40}$. **Left**: ground truth, **Middle**: STOF approach, **Right**: spatial OF approach.

The perforation in the middle of the sheet metal sample led to more complex deformation states. In contrast to the regular compression test, this test also showed inhomogeneous motion patterns. With the knowledge of the displacements and strains that are underlying the images, it is possible to compare the performance of the spatially regularised method according to Hewer and the proposed spatio-trajectorial method. The qualitative comparison of the calculated data of the proposed approach with the simulation results is depicted in Figure 3 and Figure 4.

Figure 3 depicts the horizontal and vertical displacements. On the left of both subplots, the ground-truth is shown. The results of the proposed STOF method are depicted in the middle figure, while the displacement field calculated by the purely spatial approach according to Hewer is visualised on the right. For both optical flow methods, the magnitudes and spatial distribution of the displacements agree well with the ground truth data. However, the displacement data of the spatio-temporally regularised method turned out to be significantly smoother than those of the optical flow method of Hewer. This may be due to the integration of additional image information by the additional trajectorial regularisation.

Figure 4a shows the ground truth strains, the strain distribution of the proposed approach, as well as of Hewer’s optical flow method from left to right. The spatial distribution of the strains was clearly better reproduced by the newly presented STOF regularised approach. The magnitude of the calculated strain was slightly reduced compared to the ground truth for the presented approach. Compared to the approach according to Hewer, however, it was much closer to reality, and even the secondary pattern can be resolved. Boundary artefacts occurred at the image edges.

The displacements and strains were composed of the incremental displacements and strains that occurred between the individual frames. Accordingly, the strains and strain rates (incremental strains in time) did not differ for the first time step. Nevertheless, in order to assess the smoothness of the incremental strains in time, the strain accelerations are shown in Figure 4b. While the strain accelerations calculated via the proposed approach roughly resembled the strain accelerations depicted in the simulation data, again even the secondary pattern, hardly any structure was discernible in the strain accelerations calculated via Hewer’s optical flow approach.

Further, in order to enable a quantitative evaluation, a generally applicable error metric or performance indicator is required. As a performance indicator, the concept of the angular error:

(10) $$AE=\cos^{-1}\left(\frac{1+u\times u_{GT}+v\times v_{GT}}{\sqrt{1+u^{2}+v^{2}}\cdot \sqrt{1+u_{GT}^{2}+v_{GT}^{2}}}\right)$$

proposed by [28] for the evaluation of optical flow methods, was used. This concept calculates the angular error between the calculated displacements w=(u,v) and the actually existing displacements $w_{GT}=\left(u_{GT},v_{GT}\right)$. The index GT references the ground truth displacements.

**Figure 5:**
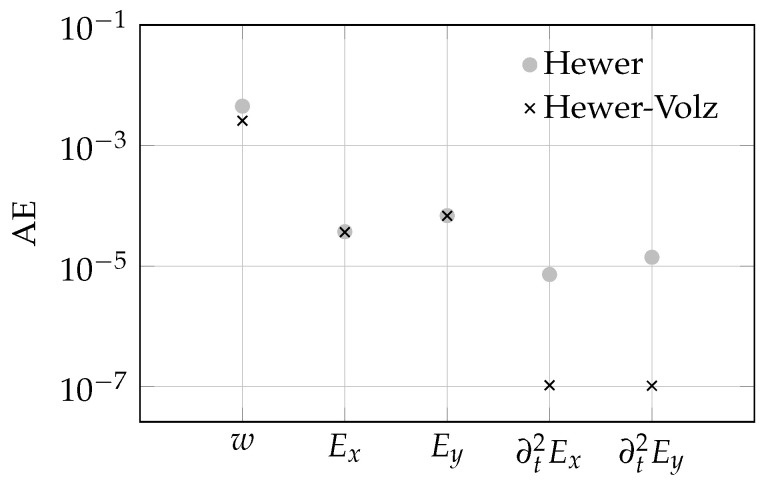
Mean angular errors (AEs) of the displacements, strains, and strain rates for the spatial OF approach (Hewer) and the proposed STOF approach (Hewer–Volz).

Figure 5 summarises the mean absolute error metrics of the angular error for the displacements, strains, and strain accelerations of the purely spatially regularised method according to Hewer et al. [12], as well as the proposed STOF regularised method. For the purely spatial variation method, the angular errors of the quantities determined under the weighting factors $\alpha_{i}=2000,\;i\in \left\{1,2,3\right\}$ are shown. These weights were also retained for the spatio-temporal variation approach, while the factor ζ of the trajectorial regularisation term was chosen as 10.

Regarding the angular errors of the displacements, the STOF approach performed better than the purely spatial regularisation. This may be due to the added trajectorial regularisation of the displacement field, integrating additional information in the calculation process and, hence, making the results more robust.

Since the two methods calculate the displacement gradients in addition to the displacements by default, these do not have to be derived by numerical differentiation for strain derivation. Hence, they do not display the numerical noise amplification caused by the operation of finite differences compared to DIC or standard optical flow approaches. Considering the angular errors of the strain fields, the two optical flow methods are on par. Since only the displacements are regularised in a trajectorial manner, the calculation procedure of the strain fields does not differ for both approaches except depending on the prior calculated displacement fields. That there is a difference in the calculated strain fields despite almost coinciding angular errors $AE(E_{x})$ and $AE(E_{y})$ can be seen considering the strain distributions of Figure 4, as well as the angular errors of the corresponding strain accelerations. These fields were derived from the strain data by numerical differentiation in the trajectorial direction. In fact, the strain data of the combined STOF approach were far smoother in the temporal direction than the purely spatial approach, resulting in less noise amplification due to the numerical differentiation procedure and, hence, more reliable estimates.

## 5. Experiments

After the optical flow method was evaluated in the previous section by the use of validation data, it was applied to real experimental image data. A shear separation process, as used, for example, for sheet metal separation in the automotive industry, was analysed. The aim of the investigation was to determine the displacements, strains, and strain rates underlying the image data.

### 5.1. Experimental Setup

**Figure 6:**
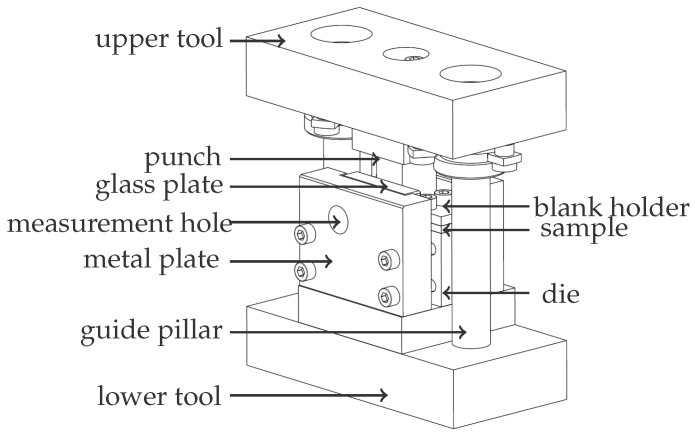
Shear cutting tool for plane strain deformation measurement.

Before presenting the results, the generation of the experimental image data is briefly described. For a more detailed description of the experimental setup, testing, and mechanisms, refer to [7]. As the sample, a piece of sheet metal of a thickness of 4 mm was used. The dimensions of the width and height were adapted to the installation geometry of the blank holder of the shear apparatus, as shown in Figure 6. Due to the grinding process, the surface of the sheet metal sample was strongly marked by grinding marks in the vertical direction. Although the grinding marks caused a strongly varying contrast in the horizontal direction, the intensity values were almost identical in the vertical direction. This made the displacement calculation robust in the horizontal direction, but left the estimation quite difficult in the vertical direction. In addition, reflections at grinding grooves that appear as local intensity differences in the image data can lead to incorrect calculations of the motion information. For these reasons, the observed sample surface was polished, and its grain size, which resembles a natural speckle pattern, was enhanced in an etching step. When the sample preparation is complete, it can be clamped into the shear cutting device shown in Figure 6.

The shearing device consisted of an upper and a lower tool part. Their positioning with respect each other was achieved by two lateral guide columns. Clamped in a press, the force was applied to the sheet sample via these columns. As the force on the upper tool part increased, it was progressively lowered onto the lower tool part. The punch also moved with it and pressed the sheet metal sample, which was fixed with a blank holder, into the die. This process can be observed through the measuring hole in the metal plate. In addition, a glass plate was inserted between the metal plate and the sheet metal sample in order to be able to observe the plain strain state in the area of the measuring hole [2]. The cross-section of the shearing sheet metal was recorded by a high-speed camera over the duration of the test with a recording frequency of 2000 Hz through the measuring hole, through which the sample was simultaneously illuminated.

**Figure 7:**
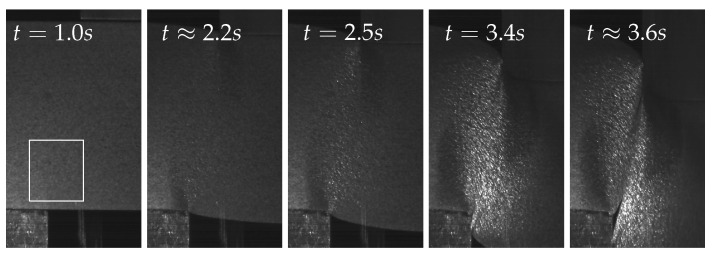
Image sequence of the sheet metal deformation and separation process, indicating the analysed region of interest in white.

Figure 7 shows some exemplary images over the course of the deformation of the sheet metal sample. While the first image shows a purely elastic deformation of the material, a plastic deformation already occurred at time step t=2.5 in the second image. In Image 3 of the time series, the force maximum was exceeded by that time, and the beginning of a crack can be observed starting from the die. This crack continued to propagate until the deformation capacity of the material was exhausted and the material cohesion was lost. The last image of the time series visualises the condition just after fracture.

**Figure 8:**
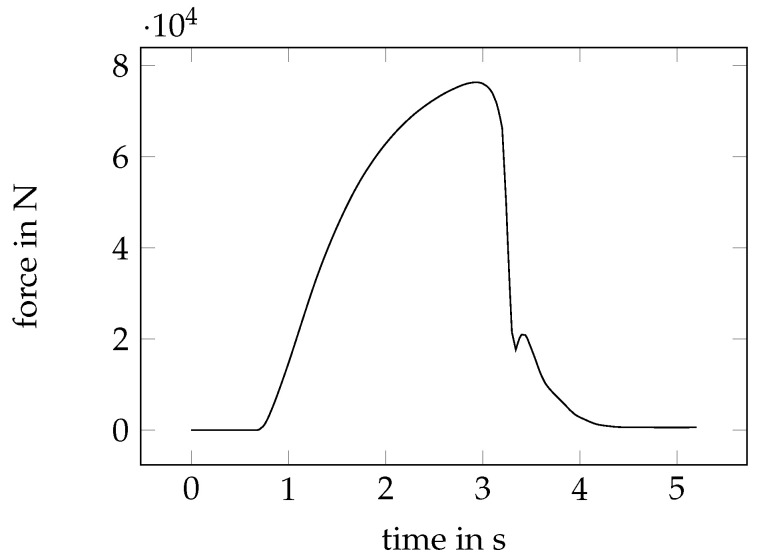
Force–time curve of the shear cutting experiment.

The corresponding force–time curve is visualised in Figure 8. At the beginning, a pre-force was applied. The actual shear process started from a time step of t=0.68 s with a constant speed of 1 mm/s. The initially linear increase of the elastic range changed into a plastic deformation. At t≈3 s, the maximum force was reached and fracture occurred. Under subsequent analysis of the fracture surface, a secondary clean cut can be observed, which explained the second local force maximum.

### 5.2. Experimental Results

In this section, the results of applying the combined spatial–trajectory approach to the experimental data of a shear cutting experiment are given. The parameters specified in Table 1 were used. They were proven to be appropriate based on a convergence analysis of the validation data. Every tenth image of it was evaluated with the proposed flow algorithm.

**Figure 9:**
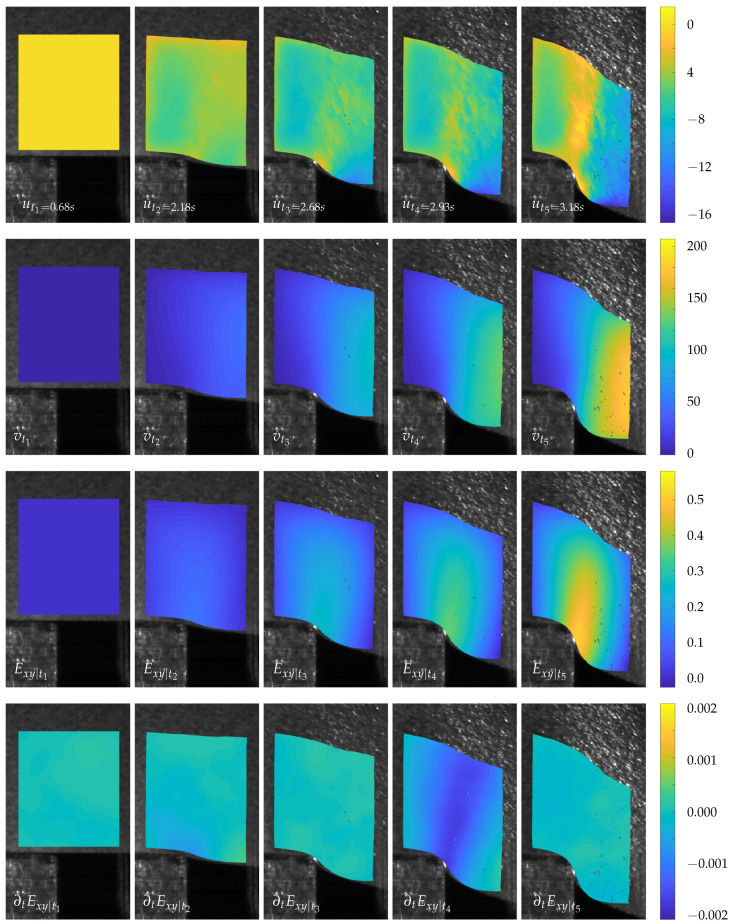
Displacement, strain, and strain rate fields of selected time steps, calculated via the STOF approach.

The displacement fields calculated under this parameter specification are shown in the first two rows of Figure 9, with the first line depicting the horizontal displacements *u* and the second line the vertical ones *v*.

For the vertical displacements, only the right material region under the stamp experienced the displacement, while the left part, which was fixed by the blank holder, did not move vertically at all. The material region located in the vicinity of the shear cutting clearance blended in, gradually increasing the vertical motion to the right. The horizontal displacement essentially took place in the area of the cutting clearance and can be separated into two regions. The material region to the right of the later crack propagation line was displaced toward the punch, while the left region was displaced toward the blank holder. The shear strains, which represent the strains dominating the shear process, are shown in Line 3. The shear band, which is characteristic for the shear process, was formed in the region of the cutting gap, starting from the die edge. The corresponding strain rate fields are depicted in the lower line of the figure. They are most dominant in the second image, capturing the state of the inflection point in the force–time diagram.

Overall, the calculated displacement, strain, and strain rate fields were reasonable and quite smooth in their appearance. Only the boarder areas suffered from calculation artefacts.

## 6. Summary

An optical flow method was implemented to improve the optical deformation analyses in the field of experimental mechanics. The primary goal was, in addition to the displacement calculations, especially the robust estimation of the strain and strain rates, which commonly suffers from noise amplification due to numerical differentiation. A higher-order purely spatial variation approach served as a starting point. The order of the spatial regularisation term was fixed to three. In order to establish the smoothness in a temporal manner in addition to the spatial smoothness, the spatial approach was supplemented by a trajectorial variation of second-order.

The variation method was evaluated on the basis of artificially generated validation data of a perforated flat tensile specimen. Further, the optical flow method was successfully applied to a real high-speed image sequence of a shear cutting experiment to estimate the underlying complex deformation pattern.

## 7. Conclusions

Overall, by the integration of a trajectorial variation, a significant improvement of the displacement computations was achieved compared to the purely spatial variation optical flow approach. Although the angular errors of the strains of the two optical flow methods were about on the same level, the proposed STOF variation yielded smoother results that were closer to the ground truth, both for the strain fields, as well as for the strain acceleration fields. Hence, in order to obtain meaningful strain rate fields, it is essential to ensure temporally smooth strain fields. Comparing the angular errors of the second-order rate fields, the introduction of the trajectorial variation results in a reduction of the angular errors by approximately two orders of magnitude. Hence, in order to obtain meaningful strain rate fields, it is essential to ensure temporally smooth strain fields.

## 8. Outlook

The results in a practical application already showed the power of the proposed methods; see [2,26]. It was even possible to go beyond robust strain rate computation, evaluating deformation curvatures and their rates, which are variables relevant for advanced modelling approaches in mechanics, such as strain gradient plasticity. Despite the already significantly improved results using a global optical flow method with the incorporation of a trajectorial variation, further improvements can be implemented, and regularisation may be tailored even more towards the tasks at hand. The variable *B*, for example, so far, was only adopted for regularisation purposes; however, it has a physical interpretation that may be exploited for evaluation. For instance, the presence of higher-order deformation gradients in elasticity requires the presence of a so-called interstitial power supplementing the standard stress inner power for thermodynamical consistency [34]. This supplementary power is related to interactions on the microscopic scale [35] and may be described in terms of such second-rank effects [36].

In a straightforward next step, the integration of a trajectorial variation not only with respect to the displacements, but also with respect to the displacement gradients is foreseen.

## Figures and Tables

**Table 1:** Parameter specifications of the STOF variational approach.

Parameter	Description
γ=0.5	relative weight of the BCA and GCA of $E_{d}$
α=2000	weight of spatial regulariser $E_{s}$
ζ=10	weight of trajectorial regulariser $E_{t}$
θ=0.5	weight to reduce influence of constraints of larger temporal distance
Ψ	$\Psi_{D}$: robust penaliser (ε=0.001) for $E_{d}$, $E_{s}$
	$\Psi_{C}$: Charbonnier penaliser (λ=1) for $E_{t}$
ω=1.97	SOR parameter

## Data Availability

The data presented in this study are available on request from the corresponding author.

## Abbreviations

The following abbreviations are used in this manuscript:

AE	Angular error
BCA	Brightness constancy assumption
DIC	Digital image correlation
GCA	Gradient constancy assumption
GT	Ground truth
OF	Optical flow
SOR	Successive over-relaxation
STOF	Spatio-trajectorial optical flow

## References

[1] Sharpe W. (2008). Springer Handbook of Experimental Solid Mechanics.

[2] Hartmann C., Lechner P., Volk W. (2021). In-situ measurement of higher-order strain derivatives for advanced analysis of forming processes using spatio-temporal optical flow. CIRP Ann..

[3] Nie G.Y., Bodda S.S., Sandhu H.K., Han K., Gupta A. (2022). Computer-Vision-Based Vibration Tracking Using a Digital Camera: A Sparse-Optical-Flow-Based Target Tracking Method. Sensors.

[4] Liu S., Liu D., Srivastava G., Połap D., Wozniak M. (2021). Overview and methods of correlation filter algorithms in object tracking. Complex Intell. Syst..

[5] Mekala M., Park W., Dhiman G., Park J., Jung H.Y. (2022). Deep Learning Inspired Object Consolidation Approaches Using LiDAR Data for Autonomous Driving: A Review. Arch. Comput. Methods Eng..

[6] Al-Qudah S., Yang M. (2023). Large Displacement Detection Using Improved Lucas-Kanade Optical Flow. Sensors.

[7] Hartmann C., Weiss H.A., Lechner P., Volk W., Neumayer S., Fitschen J.H., Steidl G. (2021). Measurement of strain, strain rate and crack evolution in shear cutting. J. Mater. Process. Technol..

[8] Horn B., Schunck B. (1981). Determining Optical Flow. Artif. Intell..

[9] Wedel A., Cremers D. (2011). Stereo Scene Flow for 3D Motion Analysis.

[10] Corpetti T., Memin E., Santa Cruz A., Heitz D., Arroyo G. Optical flow estimation in experimental fluid mechanics. Proceedings of the Seventh International Symposium on Signal Processing and Its Applications.

[11] Alvarez L., Castaño C.A., García M., Krissian K., Mazorra L., Salgado A., Sánchez J. (2007). Second order variational optic flow estimation. Computer Aided Systems Theory—EUROCAST 2007.

[12] Hewer A., Weickert J., Seibert H., Scheffer T., Diebels S. Lagrangian Strain Tensor Computation with Higher Order Variational Models. Proceedings of the 24th British Machine Vision Conference.

[13] Bredies K., Kunisch K., Pock T. (2010). Total Generalized Variation. SIAM J. Img. Sci..

[14] Trobin W., Pock T., Cremers D., Bischof H., Rigoll G. (2008). An Unbiased Second-Order Prior for High-Accuracy Motion Estimation. Proceedings of the DAGM 2008: Pattern Recognition.

[15] Volz S., Bruhn A., Valgaerts L., Zimmer H. Modeling temporal coherence for optical flow. Proceedings of the 2011 International Conference on Computer Vision.

[16] Fortun D., Bouthemy P., Kervrann C. (2015). Optical Flow Modeling and Computation: A Survey. Comput. Vis. Image Underst..

[17] Ding Z., Zhao R., Zhang J., Gao T., Xiong R., Yu Z., Huang T. Spatio-Temporal Recurrent Networks for Event-Based Optical Flow Estimation. Proceedings of the AAAI Conference on Artificial Intelligence 2022.

[18] Murray D., Buxton B. (1987). Scene Segmentation from Visual Motion Using Global Optimization. IEEE Trans. Pattern Anal. Mach. Intell..

[19] Nagel H. Extending the ’Oriented Smoothness Constraint’ into the Temporal Domain and the Estimation of Derivatives of Optical Flow. Proceedings of the ECCV.

[20] Zimmer H., Bruhn A., Weickert J. (2011). Optic Flow in Harmony. Int. J. Comput. Vis..

[21] Weickert J., Schnörr C. (2001). Variational Optic Flow Computation with a Spatio-Temporal Smoothness Constraint. J. Math. Imaging Vis..

[22] Brox T., Bruhn A., Weickert J. (2006). Variational motion segmentation with level sets. Proceedings of the European Conference on Computer Vision (ECCV).

[23] Black M., Anandan P. Robust dynamic motion estimation over time. Proceedings of the 1991 IEEE Computer Society Conference on Computer Vision and Pattern Recognition.

[24] Salgado de la Nuez A., Sánchez Pérez J. Temporal Constraints in Large Optical Flow Estimation. Proceedings of the EUROCAST, Las Palmas de Gran Canaria.

[25] Hewer A. (2013). A Generic Framework For Smoothness Terms Of Arbitrary Order.

[26] Hartmann C., Volk W., Daehn G., Cao J., Kinsey B., Tekkaya E., Vivek A., Yoshida Y. (2021). Full-Field Strain Measurement in Multi-stage Shear Cutting: High-Speed Camera Setup and Variational Motion Estimation. Forming the Future.

[27] Werlberger M., Trobin W., Pock T., Wedel A., Cremers D., Bischof H. (2009). Anisotropic Huber-L1 Optical Flow. Proceedings of the BMVC.

[28] Brox T., Bruhn A., Papenberg N., Weickert J., Pajdla T., Matas J. (2004). High Accuracy Optical Flow Estimation Based on a Theory for Warping. Proceedings of the Computer Vision—ECCV 2004.

[29] Papenberg N., Bruhn A., Brox T., Didas S., Weickert J. (2006). Highly Accurate Optic Flow Computation with Theoretically Justified Warping. Int. J. Comput. Vis..

[30] Morton K., Mayers D. (2005). Numerical Solution of Partial Differential Equations: An Introduction.

[31] Baker S., Scharstein D., Lewis J.P., Roth S., Black M.J., Szeliski R. (2011). A Database and Evaluation Methodology for Optical Flow. Int. J. Comput. Vis..

[32] Geiger A., Lenz P., Urtasun R. Are we ready for autonomous driving? The KITTI vision benchmark suite. Proceedings of the 2012 IEEE Conference on Computer Vision and Pattern Recognition.

[33] Hartmann C., Wang J., Opristescu D., Volk W. (2018). Implementation and evaluation of optical flow methods for two-dimensional deformation measurement in comparison to digital image correlation. Opt. Lasers Eng..

[34] Dunn J., Serrin J. (1985). On the thermomechanics of interstitial working. Arch. Ration. Mech. Anal..

[35] Capriz G. (1985). Continua with latent microstructure. Arch. Ration. Mech. Anal..

[36] Mariano P.M. (2017). Second-neighbor interactions in classical field theories: Invariance of the relative power and covariance. Math. Methods Appl. Sci..

